# Study on the Performance and Mechanisms of High-Performance Foamed Concrete

**DOI:** 10.3390/ma15227894

**Published:** 2022-11-08

**Authors:** Guodong Xian, Zhe Liu, Zhen Wang, Xuejun Zhou

**Affiliations:** 1 School of Civil Engineering, Shandong Jianzhu University, Jinan 250101, China; 2 Shandong Academy of Building Research Co., Ltd., Jinan 250031, China; 3 School of Traffic & Civil Engineering, Shandong Jiaotong University, Jinan 250357, China

**Keywords:** foamed concrete, graphene oxide, compressive strength, thermal conductivity, hydration reaction, microscopic morphology

## Abstract

As a common building insulation material, foamed concrete has been widely used in engineering practice. However, the contradiction between compressive strength and thermal conductivity has become the main problem limiting the development and application of foamed concrete. Therefore, high-performance foam concrete (HPFC) with high compressive strength and low thermal conductivity was prepared by using graphene oxide (GO), fly ash, and polypropylene (PP) fiber as the main admixtures, and taking compressive strength, thermal conductivity, and microstructure as the main indices. Scanning electron microscopy, X-ray diffraction (XRD), and thermogravimetry–differential scanning calorimetry (TG-DSC) were employed to examine the mechanisms of HPFC. The results showed that when the content of fly ash was 25–35 wt%, PP fiber was 0.2–0.4 wt%, and GO was 0.02–0.03 wt%, the FC’s compressive strength increased by up to 38%, and its thermal conductivity reduced by up to 3.4%. Fly ash improved the FC’s performance mainly through filling, pozzolanic activity, and slurry fluidity. PP fiber enhanced the performance of FC mainly through bridging cracks and skeletal effects. The addition of GO had no significant impact on the type, quantity, or hydration reaction rate of the hydration products in these cement-based materials, and mainly improved the FC’s microstructural compactness through template action and crack resistance, thereby improving its performance.

## 1. Introduction

China has put forward the strategic goal of “carbon peaking and carbon neutrality” to cope with global climate change. Reducing carbon dioxide emissions and improving energy utilization rates have become the focus of the current engineering construction field [1,2,3,4,5,6,7,8]. The energy consumption of buildings accounts for ~36% of the total global energy demand and 40% of the total direct and indirect carbon dioxide emissions [9]. A considerable proportion of building energy consumption is used for heating and cooling [9,10]. Therefore, energy saving in buildings can effectively alleviate or diminish the energy shortage. The goal of developing and applying thermal insulation materials has become an essential measure for buildings’ energy conservation, because the use of such materials can effectively improve the thermodynamic performance of the structural envelope and reduce building energy consumption [11,12,13,14].

Currently, commonly used thermal insulation materials for buildings are divided into organic and inorganic thermal insulation materials. Organic thermal insulation materials have low thermal conductivity (mostly <0.05 W/(m∙K)), light weight (density mostly ~100 kg/m^3^), convenient construction, and other advantages [12,15]. Due to the large-scale application of organic thermal insulation materials, fire accidents in Beijing, Shanghai, and other places have also exposed the fatal shortcomings of these materials’ poor fire performance and high-temperature resistance, resulting in high casualties and economic losses [16]. Therefore, inorganic building insulation materials have become the focus of research on the engineering applications of building materials, and foamed concrete (FC) has gradually become the first-choice building insulation material due to its excellent comprehensive performance. FC is a porous inorganic material made of foam and concrete slurry after complete mixing. It has the advantages of light weight, heat preservation, and fire prevention, and has been widely used in engineering practice [17,18,19].

Generally speaking, the compressive strength of FC increases with its dry density, while its thermal insulation performance decreases concurrently [20,21,22]. The contradiction between compressive strength and thermal conductivity has become the main problem limiting its application. Therefore, auxiliary admixtures, such as fly ash (FA) and fiber, have been widely used in FC materials. The results have shown that the filling effect and pozzolanic impact of FA can improve FC’s structural and physical properties. Yuan et al. [23] found that FA can have more uniform contact with foam and reduce the amount of foam. More uniform paste and lower foam volume could prevent bubbles from merging. This effect reduces the pore size of foam concrete and makes its pore size distribution more uniform. She et al. [24] found that when coarse FA (FAc) was used to replace sand, the mechanical properties and freeze–thaw resistance of FC were significantly improved, and the microstructure was denser. Chung et al. [25] found that FA and silica fume can make the microstructure of FC denser and help improve FC’s compressive strength and impermeability. Fiber has the function of retarding crack development, improving compressive strength, and effectively improving FC’s durability. Amran et al. [17] found that fiber can effectively improve the mechanical properties of FC, with the greatest impact on flexural strength and impact strength. Jhatial et al. [26] found that, with a fiber content of 0.2%, FC has higher strength and lower thermal conductivity, mainly because the fiber inhibits the expansion of microcracks, improving the FC’s pore structure. However, excessive FA and fiber contents might affect the early hydration reactions and pore structure performance of FC, which would be counterproductive [23,26].

Graphene oxide (GO) has attracted extensive attention in cement-based materials due to its excellent physical, mechanical, and thermal conductivity properties [27]. Studies have shown that an appropriate amount of GO can regulate cement hydration and improve structural compactness, thereby significantly improving the mechanical properties and thermal insulation properties of cement-based materials. Pan et al. [28] found that the introduction of 0.05 wt% GO increases the compressive strength by 33% and the flexural strength by 59%. Indukuri et al. [29] found that GO causes the morphology of hydration products and cement particles to be more easily dispersed in pores, adjusting the pore structure properties of the hydration products, thereby improving their physical properties. Lv et al. [30] found that GO causes a hydrated crystal flower shape and can play the role of filling holes and cracks, making the hydration product more compact, so as to improve the physical properties of cement. However, the mechanism of GO’s effects on cement-based materials is still controversial, mainly because its effects on cement hydration reactions remain unclear. Some scholars have argued that the oxygen-containing functional groups of GO can provide adsorption sites for cement and water and have catalytic properties, promoting the hydration reactions of cement [31,32]. Other scholars hold different views, showing through many experimental studies that GO does not participate in cement hydration reactions but mainly plays a template role in cement-based materials for regulating the formation of regular microstructures in cement hydration products [30,33].

**Figure 1:**
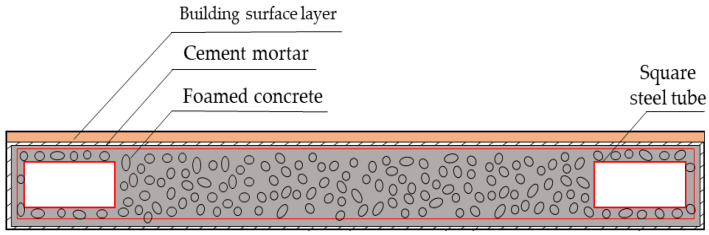
Steel frame–light steel and FC composite wall panel.

In recent years, due to improvements in energy-saving requirements in buildings and the application of steel frame–light steel and FC composite wall panels, higher standards have been put forward for FC’s mechanical properties and thermal insulation performance. The steel frame–light steel and FC composite wall panel is shown in Figure 1. The demand for high-performance foamed concrete (HPFC) in construction has become increasingly vital [34,35,36].

At present, current research mainly focuses on the study of FA, fiber, slag, and other admixtures on the physical properties and mechanisms of action of FC. There are few reports on the study of GO FC, and the effects of GO on the mechanisms of action and properties of FC remain unclear. Therefore, in this study, GO, FA, and polypropylene (PP) fiber were used as the main reinforcement materials to prepare HPFC. The compressive strength and thermal conductivity of the FC were studied, the content ranges of GO, FA, and PP fiber were determined, and the hydration process and microscopic morphology of HPFC were studied using scanning electron microscopy (SEM), X-ray diffraction spectroscopy (XRD), thermogravimetry–differential scanning calorimetry (TG-DSC), and other analytical and testing methods, to determine the mechanisms of HPFC.

## 2. Experimental Section

### 2.1. The Raw Materials

Chinese Portland cement PO 52.5 (provided by Dezhou Zhonglian Co., Ltd., Dezhou, China) was used in this research; the physical properties of the cement are presented in Table 1. FA (Dezhou Huaneng Thermal Power Plant Co., Ltd., Dezhou, China) and silica fume (Boken Silicon Material Co., Ltd., Zibo, China) were added as supplementary cementitious materials to replace a portion of the cement.

**Figure 2:**
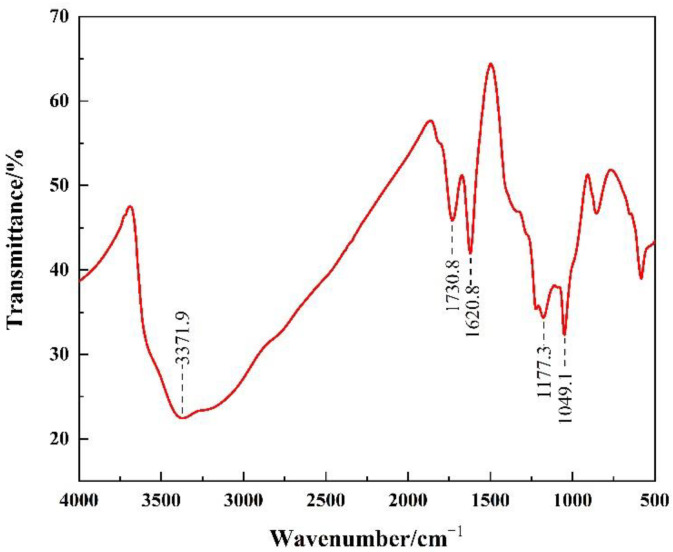
Fourier-transform infrared spectroscopic characterization of GO.

PP fiber (Xingtai New Material Co., Ltd., Jinan, China) with a length of 6–8 mm was used as a toughening material in this study. GO was another reinforcing and toughening material, with a thickness of 1.5–2 nm (Shandong Woene New Material Technology Co., Ltd., Linyi, China). The Fourier-transform infrared spectroscopic characterization of GO is shown in Figure 2.

Zhengzhou Yuke New Building Materials Machinery Co., Ltd. (Zhengzhou, China) provided the foaming agent used here. The dilution factor was 40-fold, and the density of the diluted solution was 1010 kg/m^3^.

### 2.2. Preparation Method

**Figure 3:**
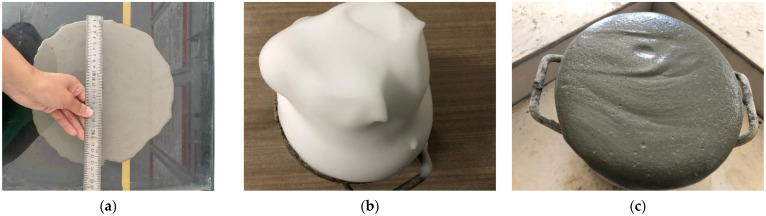
Preparation of samples: (**a**) slurry; (**b**) foam; (**c**) foamed concrete slurry.

The mixing ratio of HPFC is shown in Table 2. A physical foaming method was used to prepare HPFC for testing, and the preparation process was as follows:

(1) According to the requirements of JGJ/T 341-2014 [37], dry materials—such as cementitious materials and fibers—were stirred for 3 min. Then, water, admixture, and GO were added and mixed for 45 min to disperse the GO evenly in the slurry (Figure 3a).If there was no GO, the stirring was for 7 min.
(2) The foaming agent was prepared in advance and the foaming mechanism was used to prepare the foam (Figure 3b). The foam was added to the slurry and stirred for 3 min to form the foamed concrete slurry (Figure 3c), which was then poured into the mold. After curing for 24 h, the mold was removed and test blocks were placed in the standard curing box.
(3) It should be noted that, unless otherwise specified, the percentage sign of the mixing amount in this chapter represents the mass fraction of the cementitious material.

### 2.3. Test Methods

#### 2.3.1. Physical Performance Test

Compressive strength was determined by referring to JG/T 266-2011 [38]. The test block size was 100 mm × 100 mm × 100 mm, with 3 blocks per test group and an experimental loading speed of 1 kN/s.

Thermal conductivity was determined using GB/T 10294-2008 [39]. The test block size was 300 mm × 300 mm × 30 mm, with 2 test blocks per group.

#### 2.3.2. SEM Analysis

A JSM-7610f scanning electron microscope (SEM; JEOL Ltd., Tokyo, Japan) was used to observe the micromorphology of the HPFC samples. The experimental methods were as follows: Samples were collected from inside each block, and sample hydration was stopped in absolute ethanol. After that, the piece was dried in a drying oven to a constant weight.

#### 2.3.3. Performance Characterization of GO

The types of oxygen-containing functional groups of GO were determined using a Nicolet iS50 infrared spectrometer (Nicolet Instrument Corp., Madison, WI, USA). The experimental method was to mix the dried GO sample with potassium bromide at a ratio of 1/100 and then press the material into a tablet for measurement.

#### 2.3.4. Hydration Performance Test

The calcium hydroxide content in HPFC samples was determined using a TG/DSC-1 synchronous thermal analyzer (Mettler-Toledo International Inc., Greifensee, Switzerland). The experimental methods were as follows: a sample was placed at working temperatures of 25–1000 °C, with a heating rate of 10 °C/min and argon as a shielding gas.

Determination of the cement hydration rate and the effects of the experimental method was performed using a TAM Air isothermal calorimeter (TA Instruments Ltd., New Castle, DE, USA) within 3 days of the addition of GO, as follows: different GO contents were dispersed ultrasonically in deionized water, the GO was dispersed to the dry plastic bottles and quickly stirred well, and a calorimetric channel was added.

The Smart-Lab X-ray diffractometer (Japan Rigaku Co., Ltd., Tokyo, Japan) was used to determine the phase of HPFC, with a scanning range of 5–80°, scanning speed of 2°/min, and step size of 0.02°.

## 3. Results and Discussion

### 3.1. Effects of FA on the Properties and Microstructure of FC

**Figure 4:**
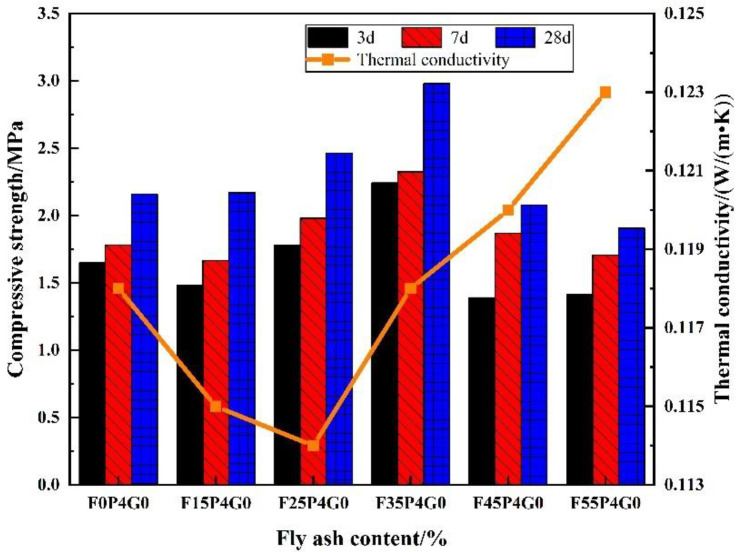
Effects of FA content on compressive strength and thermal conductivity of FC.

The effects of FA content on the compressive strength and thermal conductivity of FC were examined, and the 7 and 28 d compressive strengths of F35P4G0 reached a maximum increase of 30.9 and 38.0%, respectively, compared with F0P4G0 (Figure 4). With further increases in FA content, the compressive strength of the FC gradually decreased. F55P4G0 exhibited the lowest compressive strength; compared with F35P4G0, the 7 and 28 d compressive strength of F55P4G0 was reduced by 26.6 and 35.9%, respectively. It was not difficult to see that the FA content significantly impacted the strength of the FC.

With changes in FA content, the thermal conductivity of FC first increased and then decreased. F25P4G0 had the lowest thermal conductivity, which was 3.4% lower than that of F0P4G0. Compared with F55P4G0, the thermal conductivity of F25P4G0 was reduced by 7.3%. The thermal conductivity of F55P4G0 was 4.2% higher than that of F0P4G0.

**Figure 5:**
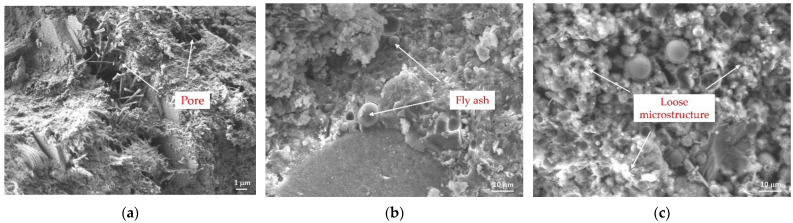
SEM images of FC with different fly ash contents: 0, 25, and 55 wt% ((**a**–**c**), respectively).

The SEM images of FC with different FA contents showed that F0P4G0, with 0% FA, had more needle-like and flake-like hydration products in the structures, which were not uniform or dense, such that there were large pores in the structure that were not conducive to structural stress (Figure 5a). In F25P4G0 (25 wt% FA), the structural needle-like and flake-like hydration products were significantly reduced (Figure 5b). Hence, the hydration products were denser, indicating that FA had a certain regulatory effect on hydration. In the process of FC preparation, the ball-shaped FA improved the fluidity of the cement slurry. FA also played a role in filling pores, which helped improve compressive strength. The F55P4G0 (55 wt% FA) microstructure was relatively loose compared with FA at 0 and 25 wt%, leading to low compressive strength of the FC (Figure 5c).

Considering the compressive strength, thermal conductivity, and microstructure of the FC samples, the performance of FC was relatively better when the fly ash content was 25–35 wt% (i.e., F25P4G0 and F35P4G0).

### 3.2. Effects of Fiber on the Performance and Microstructure of FC

**Figure 6:**
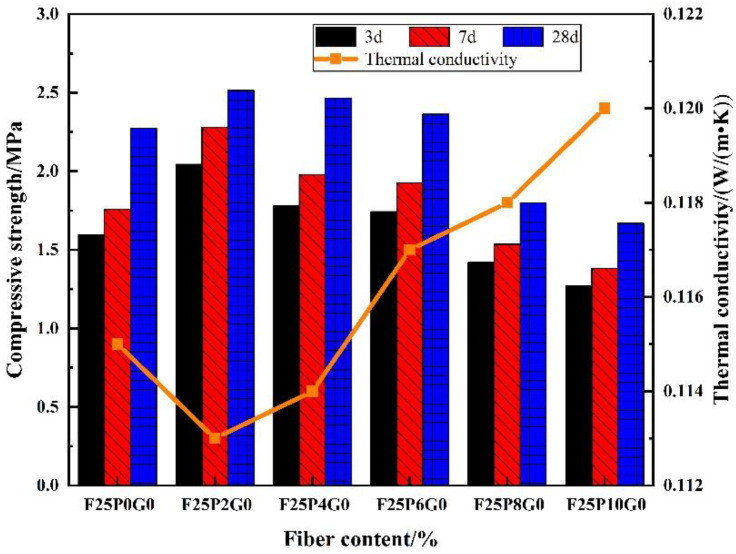
Effects of fiber content on the FC’s compressive strength and thermal conductivity.

Examination of the effects of PP fiber content on the compressive strength and thermal conductivity of FC showed that, at 7 and 28 d, the compressive strength of F25P2G0 reached a maximum—increasing by 29.5 and 10.1%, respectively, compared with F25P0G0 (Figure 6). With further increases in the fiber content, the compressive strength gradually decreased. F25P10G0 exhibited the lowest compressive strength. Compared with F25P2G0, the 7 and 28 d compressive strength of F25P10G0 was reduced by 39.5 and 33.5%, respectively.

With changes in fiber content, the thermal conductivity of FC first increased and then decreased. F25P2G0 had the lowest thermal conductivity, which was 1.7% lower than that of F25P0G0. Compared with F25P10G0, the thermal conductivity of F25P2G0 was reduced by 5.8%. The thermal conductivity of F25P10G0 was 4.3% higher than that of F25P0G0.

**Figure 7:**
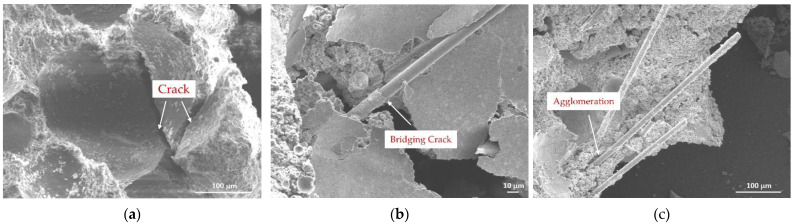
SEM images of FC with different fiber contents: 0, 0.2, and 1 wt% ((**a**–**c**), respectively).

SEM images of FC with different PP fiber contents showed that in F25P0G0, with 0 wt% fiber, due to drying shrinkage and other factors, the number of structural cracks was high, which was not conducive to structural stress (Figure 7a). F25P2G0, with 0.2 wt% fiber, played the role of bridging cracks to a certain extent, thereby reducing the number of cracks caused by drying shrinkage, while also reducing the cracks’ width (Figure 7b). Fiber randomly distributed in the slurry played a skeletal role and, according to the performance test data, greatly improved the early strength of FC. In F25P10G0, with 1 wt% fiber, the fiber was not easily evenly dispersed, and the emergence of the “agglomeration” phenomenon was not conducive to structural stress improvement (Figure 7c).

Considering the compressive strength, thermal conductivity, and microstructure of the FC samples, the performance of FC was relatively better when the fiber content was 0.2–0.4 wt% (i.e., F25P2G0 and F25P4G0).

### 3.3. Effects of GO on the Performance and Microstructure of FC

**Figure 8:**
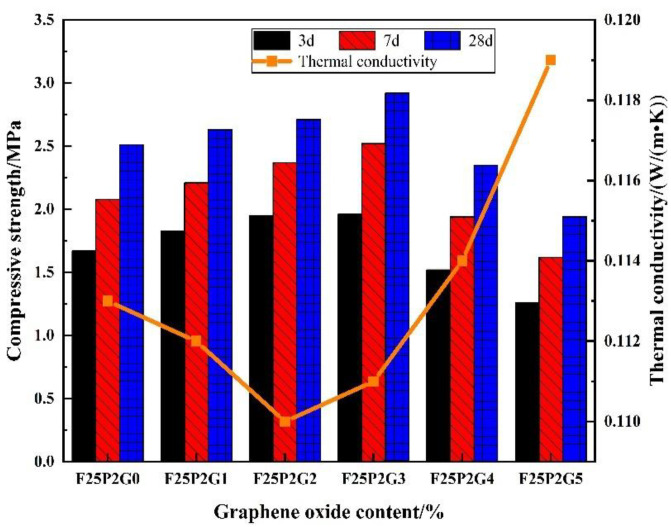
Effects of GO content on the compressive strength and thermal conductivity of FC.

Examination of the effects of GO content on the compressive strength and thermal conductivity of FC showed that, at 7 and 28 d, the compressive strength of F25P2G3 reached a maximum, increasing by 21.2 and 16.3%, respectively, compared with F25P2G0 (Figure 8). With further increases in the GO content, the compressive strength of the FC gradually decreased, where the compressive strength of F25P2G5 was the lowest—in comparison to when GO was 0.03 wt% (F25P2G3), the compressive strength at 7 and 28 d decreased by 35.7 and 33.6%, respectively. The experimental results showed that GO greatly influenced the early strength of FC.

With increased GO content, the FC’s thermal conductivity first increased and then decreased. F25P2G2 had the lowest thermal conductivity, which was 2.6% lower than that of F25P2G0. Compared with F25P2G5, the thermal conductivity of F25P2G2 was reduced by 7.6%, while the thermal conductivity of F25P2G5 was 5.3% higher than that of F25P2G0.

**Figure 9:**
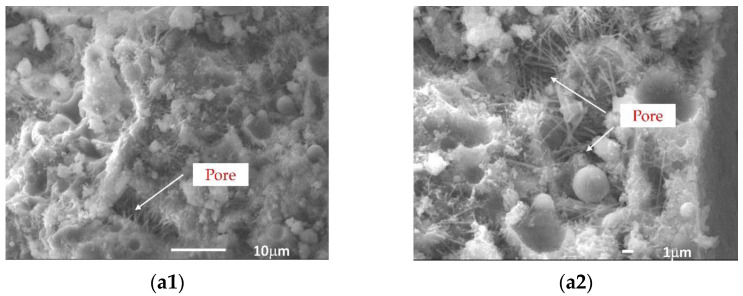

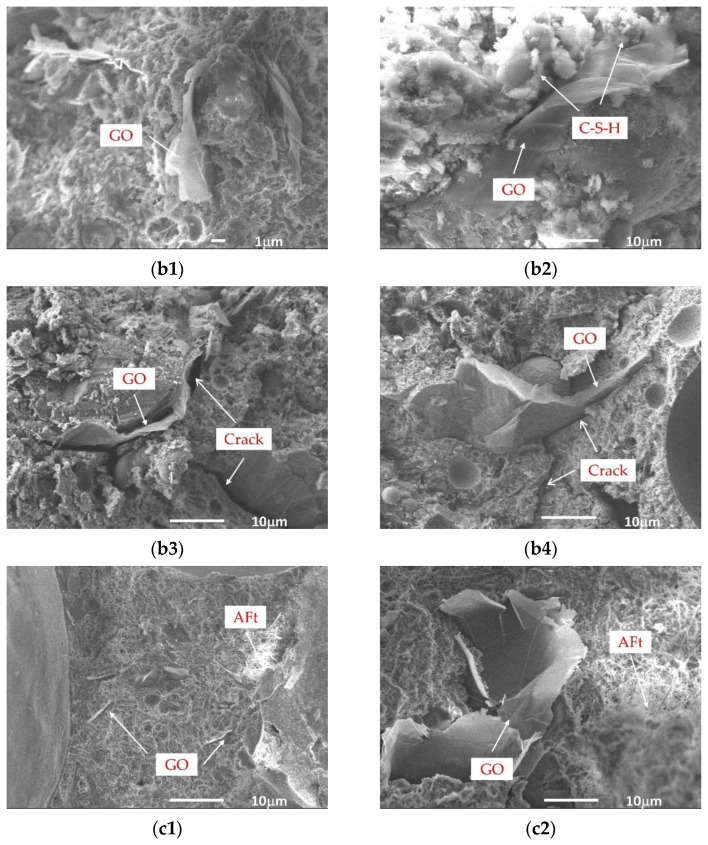
SEM images of FC with different GO contents: 0 wt% GO (**a1**,**a2**), 0.02 wt%GO (**b1**–**b4**), and 0.04 wt% GO (**c1**,**c2**).

Figure 9 shows the SEM images of FC with different GO contents. F25P2G0, with 0 wt% GO, had more acicular and flaky hydration products in the microstructure (Figure 9a). Due to bonding effects between AFt calcium hydroxide and mechanical occlusion between crystal clusters, the structure still possessed a particular strength. However, when AFt was relatively loose, there were many pores in the structure, which significantly reduced the structural compactness and, thus, was prone to adverse effects when the FC was stressed.

In F25P2G2, with 0.02 wt% GO, the hydration products near GO were stacked and intertwined to form flower-like microstructures (Figure 9(b1,b2)). There were many flocculent C-S-H gels near the GO, making the FC microstructures more compact to a certain extent, likely because the GO has a large specific surface area. The oxygen-containing functional groups on its surface can adsorb cementitious material particles and water, providing growth points for hydration products, playing a similar role in regulating hydration reactions to the template.

When the GO content was 0.02 wt%, it was seen to block the extension of cracks in these structures (Figure 9(b3,b4)). GO could have prevented the extension of cracks in the structure. As GO exhibited a curved and wrinkled morphology in the slurry, this morphology increased the adhesion between the GO and the cement matrix, thereby effectively enhancing the interfacial adhesion between the GO and the cement. The energy transmitted to the GO and the cement matrix was low when the HPFC was subjected to loading. Because GO has ultrahigh mechanical properties, most of the energy was absorbed and diffused by the GO sheets, thereby blocking the cracks. When the power transferred to the GO and the cement matrix was high, the energy absorbed and diffused by the GO sheet was limited and would continue to expand along the GO sheet. The newly generated damage would consume the remaining power and cracks would develop along the GO sheet, as observed. In the process, due to GO’s bending and wrinkled morphology in the slurry, the two components had reasonable bonding forces, ensuring GO crack resistance in the slurry and dramatically slowing the crack expansion.

In F25P2G4, with 0.04 wt% GO, the “agglomeration” phenomenon was easily observed (Figure 9(c1,c2)). The hydration product crystals near GO were stacked with one another, and the microstructure was dense. At this time, GO could also have played a template role. However, at positions far from GO, more needle-like hydration products (AFt) appeared in the structure, reducing the structural compactness. This phenomenon might have been due to the imbalance of ion concentrations caused by GO adsorption. When the GO content was high, GO could still have played a specific role in regulating hydration reactions. However, due to GO aggregation, the microstructure was dense in some regions, loose in others, and some had GO curled in the structure, which would reduce the integrity of the FC.

Considering the compressive strength, thermal conductivity, and microstructure of the FC samples, the performance of FC was relatively better when GO was 0.02–0.03 wt% (i.e., F25P2G2 and F25P2G3).

### 3.4. Effects of GO on the Hydration of FC

#### 3.4.1. X-ray Diffraction Analysis

**Figure 10:**
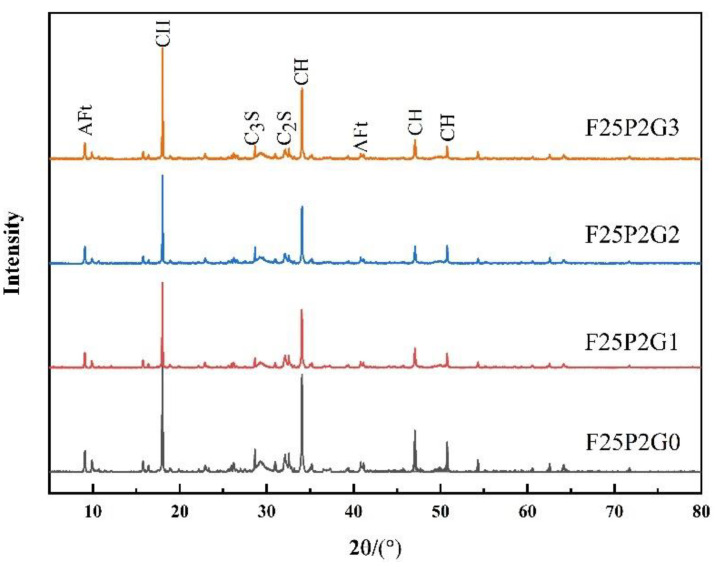
XRD spectra of FC with different GO contents.

XRD patterns of FC with different GO contents showed that the main hydration products included ettringite, calcium hydroxide, and calcium silicate hydrate (Figure 10). Due to the unfixed chemical composition of calcium silicate hydrate, no prominent diffraction peak of calcium silicate hydrate was seen. Among the other diffraction peaks, AFt represented ettringite, C-H represented calcium hydroxide, C3S represented tricalcium silicate, and C2S represented dicalcium silicate.

By comparing the XRD results of F25P2G0 with those of the F25P2G1, F25P2G2, and F25P2G3 groups, the addition of GO was seen to produce no new diffraction peaks in the XRD patterns and did not change the position of the existing diffraction peak. This phenomenon indicates that the addition of GO did not produce new hydration products, nor did it change the hydration products’ crystal types.

#### 3.4.2. Synchronizing Thermal Analysis

The main cement hydration products were calcium silicate hydrate (C-S-H), calcium sulfoaluminate hydrate (AFt, AFM), and calcium hydroxide (C-H). C-H can indirectly reflect changes in C-S-H, such that C-H content can be used to characterize the degree of hydration.

**Figure 11:**
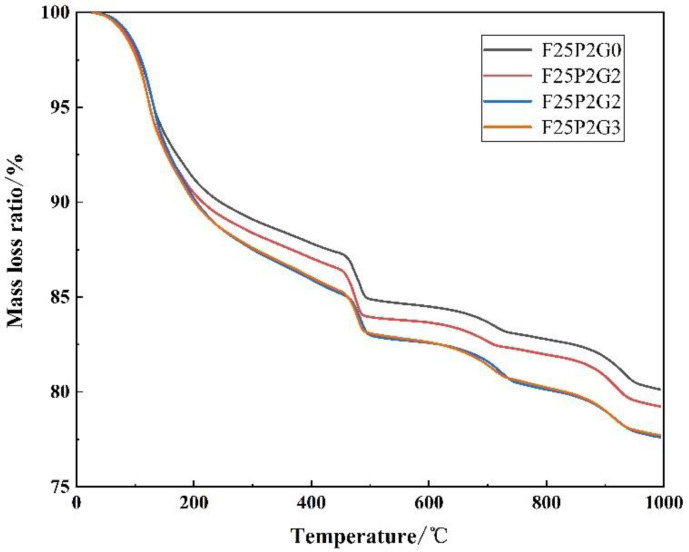
TG-DSC curves of FC with different GO contents.

No increase in or lack of endothermic peaks was observed in the four curves, indicating that no new hydration products were generated by adding GO (Figure 11). During the heating process, there were three prominent endothermic peaks: The first peak was between 105 and 200 °C, mainly caused by the decomposition of ettringite and dehydration of calcium silicate hydrate. The second peak was the endothermic peak between 450 and 550 °C, primarily reflecting the heat change caused by the decomposition of calcium hydroxide.

The third peak was the endothermic peak between 650 and 800 °C, mainly reflecting the heat change caused by the decomposition of calcium carbonate. Although the sample was taken from inside the specimen and an argon atmosphere was used during testing, it was inevitable that the sample carbonized. Therefore, carbonized C-H should be considered when calculating a sample’s C-H content. According to the mass loss of models in the heating process, the thermal decomposition of calcium hydroxide and calcium carbonate, and the molecular weight relationship of calcium hydroxide carbonization, the C-H contents of FC with different GO contents were calculated (Table 3).

After adding GO, the calcium hydroxide content of all samples showed no significant differences, indicating that GO had no noticeable effect on the cement hydration.

#### 3.4.3. Heat-of-Hydration Analysis

**Figure 12:**
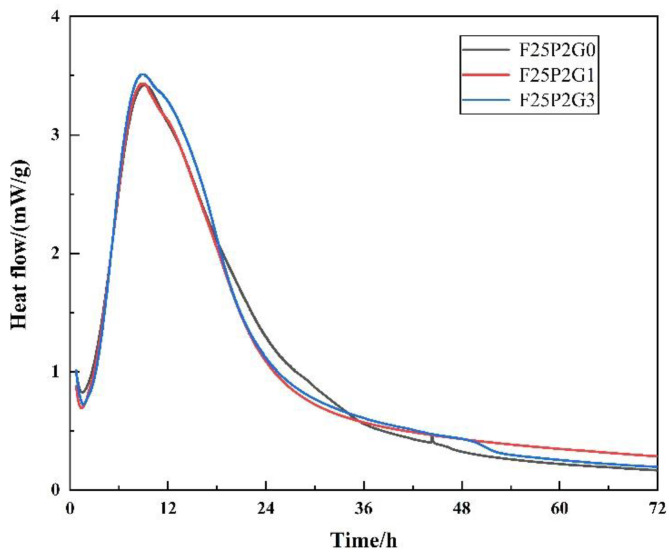
Hydration exothermic curves of FC with different GO contents.

FC’s hydration heat release curves with different GO contents (i.e., 0, 0.01, and 0.03 wt%) showed that the O–A stage (0.5–2 h) was the induction period, the A–B stage (2–12 h) was the acceleration period, the B–C stage (12–36 h) was the deceleration period, and the C–D stage (36–72 h) was the stable period (Figure 12). As external agitation was used in these tests, the exothermal peak in the initial reaction period was not reflected in the test results. The exothermic heat of the O–A segment was mainly caused by the nucleation process of the C-S-H gel, while the C-S-H gel’s growth mainly caused the A–B segment’s exothermic heat. The three curves had a high degree of coincidence in the O–A and A–B segments, and the exothermic rates were the same. After 24 h, the curve fluctuated, but the heat release rate was no different, which might have been caused by an unstable thermal balance of the instrument in the later period.

Therefore, the times for the curves to enter different hydration stages were roughly the same, as was the change in the hydration heat release rate in each set, indicating that the addition of GO had no significant effect on the hydration heat release rate of FC.

## 4. Conclusions

In this study, GO, FA, and PP fiber were selected as reinforcing materials to prepare HPFC. Its physical properties, microscopic morphology, and hydration products were analyzed. The main conclusions were as follows:

1. Compared with no admixture, when the FA content was 25–35 wt%, the FC’s compressive strength increased by up to 38.0%, while its thermal conductivity was reduced by up to 3.4%. When the PP fiber content was 0.2–0.4 wt%, the compressive strength increased by up to 10.1%, while the thermal conductivity was reduced by up to 1.7%. When the GO content was 0.02–0.03 wt%, the compressive strength increased by up to 16.3%, while the thermal conductivity was reduced by up to 2.6%. In addition, FC mixed in the above ranges had a denser microstructure.
2. FA in FC mainly filled pores and was involved in secondary hydration reactions with calcium hydroxide and other substances. At the same time, the FC slurry’s fluidity was improved because the FA was ball-shaped. The PP fiber mainly played a role in refining pores and bridging cracks in the FC. At the same time, the disorderly distribution of the fiber in the slurry also played a skeletal role and significantly improved the early strength of the FC.
3. GO mainly played the roles of templating and crack resistance in FC. Its template function was primarily due to its ultrahigh specific surface area, which adsorbed cementing material particles and water to provide growth points for hydration reactions. The crack resistance effect was mainly due to its ultrahigh mechanical properties, which absorb diffused energy, thereby slowing crack propagation.
4. The addition of GO did not produce new hydration products, and its influence on the main hydration product contents of FC was insignificant. GO also did not significantly change the FC hydration reaction rate. In short, GO had no noticeable effect on the hydration process of FC.

## Figures and Tables

**Table 1:** Physical properties of the cement.

Gravity (kg/m^3^)	Setting Time (min)		Flexural Strength (MPa)		Compressive Strength (MPa)	
	Initial Set	Final Set	3 d	28 d	3 d	28 d
3090	131	182	6.2	8.3	34.7	56.2

**Table 2:** Mix ratios of HPFC.

Specimens	Water/ Cement Ratio	Cement (kg)	Fly Ash (kg)	Silica Fume (kg)	PP Fiber (g)	Foam (kg)	Water-Reducing Agent (g)	GO (g)	Dry Density(kg/m^3^)
Fly ash + 0.04 wt% PP fiber + 0 wt% GO group									
F0P4G0	0.6	17.81	0	0.94	75	0.75	46.9	0	557.2
F15P4G0	0.6	15	2.81	0.94	75	0.75	46.9	0	532.3
F25P4G0	0.6	13.12	4.69	0.94	75	0.75	46.9	0	536.8
F35P4G0	0.6	11.25	6.56	0.94	75	0.75	46.9	0	571.1
F45P4G0	0.6	9.37	8.44	0.94	75	0.75	46.9	0	590.2
F55P4G0	0.6	7.5	10.31	0.94	75	0.75	46.9	0	605.3
25 wt% Fly ash + PP fiber + 0 wt% GO group									
F25P0G0	0.6	13.12	4.69	0.94	0	0.75	46.9	0	527.2
F25P2G0	0.6	13.12	4.69	0.94	37.5	0.75	46.9	0	529.8
F25P4G0	0.6	13.12	4.69	0.94	75	0.75	46.9	0	536.8
F25P6G0	0.6	13.12	4.69	0.94	112.5	0.75	46.9	0	550.4
F25P8G0	0.6	13.12	4.69	0.94	150	0.75	46.9	0	555.9
F25P10G0	0.6	13.12	4.69	0.94	187.5	0.75	46.9	0	576.1
25 wt% Fly ash + 0.02 wt% PP fiber + GO group									
F25P2G0	0.6	13.12	4.7	0.94	37.5	0.75	46.9	0	529.8
F25P2G1	0.6	13.12	4.7	0.94	37.5	0.75	46.9	1.9	522.3
F25P2G2	0.6	13.12	4.7	0.94	37.5	0.75	46.9	3.8	519.4
F25P2G3	0.6	13.12	4.7	0.94	37.5	0.75	46.9	5.7	527.6
F25P2G4	0.6	13.12	4.7	0.94	37.5	0.75	46.9	7.6	548.7
F25P2G5	0.6	13.12	4.7	0.94	37.5	0.75	46.9	9.5	577.8

Note: Sample F25P2G1 indicates FA content of 25%, PP fiber content of 0.02%, and GO content of 0.01%; other samples are expressed in the same manner.

**Table 3:** Mass loss of samples during heating.

Samples	450–550 °C Mass Loss (%)	650–800 °C Mass Loss (%)	Calcium Hydroxide Content (%)
F25P2G0	2.40	1.46	12.31
F25P2G1	2.48	1.37	12.50
F25P2G2	2.19	2.21	12.70
F25P2G3	2.22	2.04	12.56

## Data Availability

Not applicable.

## References

[1] Li B., Han S., Wang Y., Wang Y., Li J., Wang Y. (2020). Feasibility Assessment of the Carbon Emissions Peak in China’s Construction Industry: Factor Decomposition and Peak Forecast. Sci. Total Environ..

[2] Zhang X., Wang F. (2016). Hybrid Input-Output Analysis for Life-Cycle Energy Consumption and Carbon Emissions of China’s Building Sector. Build. Environ..

[3] Chalangaran N., Farzampour A., Paslar N., Fatemi H. (2021). Experimental Investigation of Sound Transmission Loss in Concrete Containing Recycled Rubber Crumbs. Adv. Concr. Constr..

[4] Mansouri I., Shahheidari F.S., Hashemi S.M.A., Farzampour A. (2020). Investigation of Steel Fiber Effects on Concrete Abrasion Resistance. Adv. Concr. Constr..

[5] Nafees A., Amin M.N., Khan K., Nazir K., Ali M., Javed M.F., Aslam F., Musarat M.A., Vatin N.I. (2022). Modeling of Mechanical Properties of Silica Fume-Based Green Concrete Using Machine Learning Techniques. Polymers.

[6] Khan K., Ullah M.F., Shahzada K., Amin M.N., Bibi T., Wahab N., Aljaafari A. (2020). Effective Use of Micro-Silica Extracted from Rice Husk Ash for the Production of High-Performance and Sustainable Cement Mortar. Constr. Build. Mater..

[7] Chalangaran N., Farzampour A., Paslar N. (2020). Nano Silica and Metakaolin Effects on the Behavior of Concrete Containing Rubber Crumbs. CivilEng.

[8] Farzampour A. (2017). Temperature and Humidity Effects on Behavior of Grouts. Adv. Concr. Constr..

[9] Kishore R.A., Bianchi M.V.A., Booten C., Vidal J., Jackson R. (2021). Enhancing Building Energy Performance by Effectively Using Phase Change Material and Dynamic Insulation in Walls. Appl. Energy.

[10] Marin-Montin J., Roque E., Xu Y., Šavija B., Serrano-Ruiz J.C., Montero-Chacón F. (2022). Thermomechanical Performance Analysis of Novel Cement-Based Building Envelopes with Enhanced Passive Insulation Properties. Materials.

[11] Sadineni S.B., Madala S., Boehm R.F. (2011). Passive Building Energy Savings: A Review of Building Envelope Components. Renew. Sustain. Energy Rev..

[12] Hung Anh L.D., Pásztory Z. (2021). An Overview of Factors Influencing Thermal Conductivity of Building Insulation Materials. J. Build. Eng..

[13] Yang W., Wang Y., Liu J. (2022). Optimization of the Thermal Conductivity Test for Building Insulation Materials under Multifactor Impact. Constr. Build. Mater..

[14] Majumder A., Canale L., Mastino C.C., Pacitto A., Frattolillo A., Dell’Isola M. (2021). Thermal Characterization of Recycled Materials for Building Insulation. Energies.

[15] Abu-Jdayil B., Mourad A.-H., Hittini W., Hassan M., Hameedi S. (2019). Traditional, State-of-the-Art and Renewable Thermal Building Insulation Materials: An Overview. Constr. Build. Mater..

[16] Zhou Y., Bu R., Yi L., Sun J. (2020). Heat Transfer Mechanism of Concurrent Flame Spread over Rigid Polyurethane Foam: Effect of Ambient Pressure and Inclined Angle. Int. J. Therm. Sci..

[17] Amran M., Fediuk R., Vatin N., Huei Lee Y., Murali G., Ozbakkaloglu T., Klyuev S., Alabduljabber H. (2020). Fibre-Reinforced Foamed Concretes: A Review. Materials.

[18] Tikalsky P.J., Pospisil J., MacDonald W. (2004). A Method for Assessment of the Freeze–Thaw Resistance of Preformed Foam Cellular Concrete. Cem. Concr. Res..

[19] Zhang S., Cao K., Wang C., Wang X., Wang J., Sun B. (2020). Effect of Silica Fume and Waste Marble Powder on the Mechanical and Durability Properties of Cellular Concrete. Constr. Build. Mater..

[20] Othman R., Jaya R.P., Muthusamy K., Sulaiman M., Duraisamy Y., Abdullah M.M.A.B., Przybył A., Sochacki W., Skrzypczak T., Vizureanu P. (2021). Relation between Density and Compressive Strength of Foamed Concrete. Materials.

[21] Oren O.H., Gholampour A., Gencel O., Ozbakkaloglu T. (2020). Physical and Mechanical Properties of Foam Concretes Containing Granulated Blast Furnace Slag as Fine Aggregate. Constr. Build. Mater..

[22] Dhasindrakrishna K., Pasupathy K., Ramakrishnan S., Sanjayan J. (2021). Progress, Current Thinking and Challenges in Geopolymer Foam Concrete Technology. Cem. Concr. Compos..

[23] Yuan H., Ge Z., Sun R., Xu X., Lu Y., Ling Y., Zhang H. (2022). Drying Shrinkage, Durability and Microstructure of Foamed Concrete Containing High Volume Lime Mud-Fly Ash. Constr. Build. Mater..

[24] She W., Du Y., Zhao G., Feng P., Zhang Y., Cao X. (2018). Influence of Coarse Fly Ash on the Performance of Foam Concrete and Its Application in High-Speed Railway Roadbeds. Constr. Build. Mater..

[25] Chung S.-Y., Kim J.-S., Lehmann C., Stephan D., Han T.-S., Elrahman M.A. (2020). Investigation of Phase Composition and Microstructure of Foamed Cement Paste with Different Supplementary Cementing Materials. Cem. Concr. Compos..

[26] Jhatial A.A., Goh W.I., Mohamad N., Rind T.A., Sandhu A.R. (2020). Development of Thermal Insulating Lightweight Foamed Concrete Reinforced with Polypropylene Fibres. Arab. J. Sci. Eng..

[27] Yuan X., Niu J., Zeng J., Jing Q. (2018). Cement-Induced Coagulation of Aqueous Graphene Oxide with Ultrahigh Capacity and High Rate Behavior. Nanomaterials.

[28] Pan Z., He L., Qiu L., Korayem A.H., Li G., Zhu J.W., Collins F., Li D., Duan W.H., Wang M.C. (2015). Mechanical Properties and Microstructure of a Graphene Oxide–Cement Composite. Cem. Concr. Compos..

[29] Indukuri C.S.R., Nerella R., Madduru S.R.C. (2019). Effect of Graphene Oxide on Microstructure and Strengthened Properties of Fly Ash and Silica Fume Based Cement Composites. Constr. Build. Mater..

[30] Lv S., Liu J., Sun T., Ma Y., Zhou Q. (2014). Effect of GO Nanosheets on Shapes of Cement Hydration Crystals and Their Formation Process. Constr. Build. Mater..

[31] Lin C., Wei W., Hu Y.H. (2016). Catalytic Behavior of Graphene Oxide for Cement Hydration Process. J. Phys. Chem. Solids.

[32] Sharma S., Kothiyal N.C., Chitkara M. (2016). Enhanced Mechanical Performance of Cement Nanocomposite Reinforced with Graphene Oxide Synthesized from Mechanically Milled Graphite and Its Comparison with Carbon Nanotubes Reinforced Nanocomposite. RSC Adv..

[33] Peng H., Ge Y., Cai C.S., Zhang Y., Liu Z. (2019). Mechanical Properties and Microstructure of Graphene Oxide Cement-Based Composites. Constr. Build. Mater..

[34] Xu Z., Chen Z., Yang S. (2018). Seismic Behavior of Cold-Formed Steel High-Strength Foamed Concrete Shear Walls with Straw Boards. Thin-Walled Struct..

[35] Prabha P., Marimuthu V., Saravanan M., Palani G.S., Lakshmanan N., Senthil R. (2013). Effect of Confinement on Steel-Concrete Composite Light-Weight Load-Bearing Wall Panels under Compression. J. Constr. Steel Res..

[36] Othuman Mydin M.A., Wang Y.C. (2011). Structural Performance of Lightweight Steel-Foamed Concrete–Steel Composite Walling System under Compression. Thin-Walled Struct..

[37] (2014). Technical Specification for Application of Foamed Concrete.

[38] (2011). Foamed concrete.

[39] (2018). Protective Hot plate Method for the Determination of Steady Thermal Resistance and Related Characteristics of Thermal Insulation Materials.

